# Obstetric outcome in donor oocyte pregnancies: a matched-pair analysis

**DOI:** 10.1186/1477-7827-10-42

**Published:** 2012-06-06

**Authors:** Dominic Stoop, Miriam Baumgarten, Patrick Haentjens, Nikolaos P Polyzos, Michel De Vos, Greta Verheyen, Michel Camus, Paul Devroey

**Affiliations:** 1 Centre for Reproductive Medicine, Universitair Ziekenhuis Brussel, Vrije Universiteit Brussel, Laarbeeklaan 101, B-1090, Brussels, Belgium

**Keywords:** Oocyte donation, Pregnancy outcome, Pregnancy-induced hypertension, First trimester bleeding

## Abstract

**Background:**

To investigate the obstetrical and perinatal impact of oocyte donation, a cohort of women who conceived after OD was compared with a matched control group of women who became pregnant through in vitro fertilisation with autologous oocytes (AO).

**Methods:**

A matched-pair analysis has been performed at the Centre for Reproductive Medicine of the UZ Brussel, Dutch speaking Free University of Brussel. A total of 410 pregnancies resulted in birth beyond 20 weeks of gestation occurring over a period of 10 years, including 205 oocyte donation pregnancies and 205 ICSI pregnancies with autologous oocytes (AO). Patients in the OD group were matched on a one-to-one basis with the AO group in terms of age, ethnicity, parity and plurality. Matched groups were compared using paired t-tests for continuous variables and McNemar test for categorical variables. A conditional logistic regression analyses was performed adjusting for paternal age, age of the oocyte donor, number of embryos transferred, and singleton/twin pregnancy.

**Results:**

Oocyte donation was associated with an increased risk of pregnancy induced hypertension (PIH) (matched OR: 1.502 CI: 1.024-2.204), and first trimester bleeding (matched OR: 1.493 CI: 1.036-2.15). No differences were observed between the two matched groups with regard to gestational age, mean birth weight and length, head circumference and Apgar scores.

**Conclusions:**

Oocyte donation is associated with an increased risk for PIH and first trimester bleeding independent of the recipients’ age, parity and plurality, and independent of the age of the donor or the partner. However, oocyte donation has no impact on the overall perinatal outcome.

## Background

Oocyte donation (OD) has been introduced in 1984 to allow women with ovarian insufficiency to become pregnant [1]. The success of the technique led to a broadening of scope of the treatment to include indications of repeated IVF failure, advanced maternal age or inheritable disease [2]. Today, OD has become well established with thousands of children born worldwide annually. As with any other reproductive techniques, assessment of possible associated obstetric and perinatal risk remains of paramount importance.

Several authors have reported on the obstetrical and perinatal outcome after oocyte donation. Although these case reports and series have shown varying results, the most consistently reported complications are a high incidence of pregnancy induced hypertension (PIH) and first trimester bleeding [3-7]. Most authors concluded that pregnancies after oocyte donation need to be considered as high risk pregnancies, however overall perinatal outcomes are considered favourable. [5-7].

Pregnancies after oocyte donation represent a unique group of pregnancies because they are achieved with an immunologically foreign embryo. This may underlie the observed increased obstetrical and perinatal risk associated with these pregnancies. However, obstetrical risk factors such as advanced maternal age, primiparity and multiple pregnancies tend to be more common in this population. Moreover, there is a higher incidence of potential obstetrical risk factors that necessitate oocyte donation treatment, such as previous pelvic radiation, immunologic disorders or severe endometriosis [8-10].

It remains unclear to which extent the increased obstetrical and perinatal risks can be attributed to immunological mechanisms or to the aforementioned confounding risk factors.

We therefore performed a retrospective analysis to investigate the impact of oocyte donation on the obstetric and perinatal outcome by comparing with a strictly matched control group. On a one-to-one basis, the oocyte recipient cohort was matched for age, ethnicity, parity and plurality, with a cohort of women who underwent in-vitro fertilisation treatment. Using this methodology we were able to minimise confounding bias and to investigate the effect of both oocyte donation and the underlying medical condition.

## Methods

### Study design

At the Dutch-speaking Brussels Free University, data collection with regard to the obstetrical and perinatal outcomes after oocyte donation and after ART have long been integrated into the clinical programme of the fertility centre [11]. All pregnancies that occurred between January 1999 and December 2008, that had been obtained after oocyte donation and resulted in offspring after more than 20 weeks of gestation, were included in this study. Matched controls were selected from the patient population that underwent in-vitro fertilisation with autologous oocytes during the same period. All pregnancies in oocyte recipients and in controls were conceived after intra cytoplasmic sperm injection (ICSI). Oocyte recipients underwent embryo transfer in natural cycles or in artificial cycles supplemented with a daily dose of 1600 mg micronised progesterone and 10 mg oestradiol valerate until 12^th^ weeks gestation. The individually matched controls were selected from a total of 3707 ICSI pregnancies reaching 20 weeks of gestation. Pregnancies after pre-implantation genetic diagnosis (PGD) or after testicular sperm extraction (TESE) or use of donor sperm were not included in this study. The study was approved by the committe for ethics in medical research of the Free University of Brussels (12-5-2010; 2010/106; B.U.N.14320108637).

### Data collection

For all pregnancies, written data regarding obstetrical and neonatal outcome were collected. As a routine procedure, a questionnaire is send to all patients with an ongoing pregnancy at seven weeks of gestation and to their obstetrician around the estimated delivery date. On average, 95% of the questionnaires are returned, while another 5% of the patients or their gynaecologists need to be contacted by phone in order to retrieve the outcome data. Obstetrical complications, perinatal data, including gestational age, mode of delivery, birth weight, Apgar scores, presence or absence of malformations and neonatal problems were registered. If any problem was mentioned, detailed information was requested from the paediatrician in charge.

### Matching procedure

Matching was performed for maternal age (+/− 12 months), parity (nulliparity or multiparity), plurality (singleton or twin) and maternal ethnicity. Matching for the gender of the child was only performed in singleton pregnancies. Where multiple matching options existed, case–control pairs with concordant delivery dates were selected. None of the oocytes or embryos underwent cryopreservation prior to embryo transfer.

### Definitions

Pregnancy-induced hypertension (PIH) was defined as blood pressure (BP) levels > 140/90 mm Hg on two or more occasions at least 6 h apart, without proteinuria, after 20 weeks. Pre-eclampsia: repeated BP levels ≥ 140/90 mm Hg with proteinuria ≥ 0.3 g/day after 20 weeks of gestation. Gestational age: calculated from the day of oocyte aspiration, which was defined as day 14 of the cycle. Stillbirth: intrauterine or intrapartum death of a child born with a gestational age ≥ 20 weeks or with a birth weight of ≥ 500 g. Preterm birth: delivery before 37 completed weeks of gestation. Preterm premature rupture of the membranes (pPROM) was defined as rupture of the membranes before 37 weeks of gestation in the absence of uterine contractions. Perinatal mortality: number of intrauterine or intrapartum deaths and neonatal deaths < 7 days after birth per 1000 children born with a gestational age of ≥ 20 weeks. Low birth weight: < 2500 g at birth. Very low birth weight: < 1500 g at birth.

### Statistical analysis

Categorical variables are presented as numerator and denominator values (%), and continuous variables as mean (SD, standard deviation) for each group of interest. Paired data from the oocyte donation pregnancies and their matched controls were analysed using McNemar and paired t-tests for categorical and continuous variables, respectively. Multivariable modelling was conducted using conditional logistic regression adjusting for paternal age, age of the oocyte donor, the number of embryos transferred, and singleton/twin pregnancy. Pairs with missing outcome data were not included in our matched analyses. All data were analysed using PASW version 17.0 for windows (SPSS Inc., Chicago, IL).

## Results

### Characteristics of the oocyte recipients and their matched controls

Overall, the oocyte donation programme resulted in the birth of 375 children out of 294 pregnancies (see Additional file 1: Table S1). Among pregnancies that reached 20 weeks of gestation, 97.9% resulted in a live birth. The multiple birth rate was 30.3% (89/294). Singleton pregnancies ended in 2.0% of cases with a stillbirth, while the stillbirth rate among twin pregnancies was 2.3%. None of the pregnancies was ended by an elective interruption. Additional file 1: Table S1 lists further characteristics of this oocyte recipient cohort of 294 OD pregnancies.

An appropriate match was found for 205 OD-pregnancies resulting in 262 live births. Table 1 lists characteristics of this oocyte recipient cohort of 205 OD pregnancies, and their 205 AO individually matched controls. Although no matching was performed for paternal age, body mass index (BMI) and smoking behaviour, these parameters did not differ between pregnancies conceived with donated versus those conceived with autologous oocytes. The mean age of the oocyte donors of 30.7 years was significantly younger (almost 6 years) than the mean age of the oocyte recipient and their matches.

**Table 1:** Characteristics of the oocyte recipients and their individually matched controls

	**Available data (%)**	**All pregnancies (n = 205)**			**Singletons (n = 148)**			**Twins (n = 57)**		
		**DO**	**AO**	**P**	**DO**	**AO**	**P**	**DO**	**AO**	**P**
Age										
Recipient (DO) or ICSI patient (AO) (%; SD)	410/410	36.0	36.0	0.37	36.3	36.2	0.24	35.4	35.4	0.66
	(100)	(4.5)	(4.5)		(4.5)	(4.5)		(4.4)	(4.4)	
Donor	406/410	30.7	36.1	<0.001	31.0	36.3	<0.001	29.8	35.4	<0.001
(%; SD)	(99)	(4.4)	(4.4)		(4.3)	(4.5)		(4.6)	(4.4)	
Partner	400/410	38.1	39.0	0.90	38.3	39.1	0.23	37.6	38.9	0.19
(%; SD)	(97.5)	(6.4)	(6.7)		(6.2)	(6.7)		(6.9)	(6.5)	
Parity	410/410	0.23	0.23	0.78	0.2	0.2	0.32	0.2	0.3	0.66
(%; SD)	(100)	(0.5)	(0.5)		(0.5)	(0.5)		(0.5)	(0.5)	
Ethnicity	410/410	202/205	202/205	1	146/148	146/148	1	55/57	55/57	1
(% Caucasian mothers)	(100)	(98.5)	(98.5)		(98.6)	(98.6)		(96.5)	(96.5)	
Sex baby	524/524	129/262	123/262	0.52	66/148	66/148	1	63/114	57/114	0.52
(% male)	(100)	(49.2)	(46.9)		(44.6)	(44.6)		(55.3)	(50.0)	
BMI	222/410	23.3	23.2	0.92	23.7	23.1	0.35	22.1	23.5	0.14
(%; SD)	(54.1)	(4.4)	(3.2)		(4.7)	(3.4)		(3.4)	(3.6)	
Smoking	187/410	5/37	6/37	1	5/31	6/31	1	0	0	1
(%; SD)	(45.6)	(13.5)	(16.2)		(16.1)	(19.3)				

### Obstetrical outcome after oocyte donation versus autologous matched controls

The incidence of first trimester vaginal bleeding and pregnancy-induced hypertension was significantly higher in pregnancies conceived with donated oocytes compared to matched controls with autologous oocytes: 20.6% vs. 10.3% (*P* value for McNemar test =0.005) and 19.1% vs. 8.3% (*P* = 0.002), respectively (Table 2). However, no significant difference was found in singleton pregnancies with regard to these obstetrical complications. No difference was observed in the incidence of nausea or in the incidence of hospital admission for hyperemesis. Although the observed incidences for complications such as pre-eclampsia (11.8% vs. 6.4%), HELLP syndrome (0.98% vs. 0.59%) or gestational diabetes (7.4% vs. 3.4%) were almost twice as high in pregnancies after oocyte donation these differences were not statistically significantly different.

**Table 2:** Obstetrical outcome in pregnancies with donated oocytes versus individually matched pregnancies with autologous oocytes

	**All pregnancies**			**Singletons**			**Twins**		
	**DO**	**AO**	P	**DO**	**AO**	**P**	**DO**	**AO**	**P**
	**n = 205 (%)**	**n = 205 (%)**		**n = 148 (%)**	**n = 148 (%)**		**n = 57 (%)**	**n = 57 (%)**	
Vaginal bleeding									
1^st^ trimester	42/204	21/204	0.005	32/148	20/148	0.08	10/57	1/57	0.01
	(20.6)	(10.3)		(21.8)	(13.6)		(17.5)	(1.8)	
2^nd^ trimester	5/205	6/204	1	3/148	5/147	0.73	2/57	1/57	1
	(2.4)	(2.9)		(2)	(3.4)		(3.5)	(1.8)	
3^rd^ trimester	3/205	3/205	1	2/148	3/148	1	1/57	0/57	1
	(1.5)	(1.5)		(1.4)	(2)		(1.8)	(0)	
Nausea and vomiting									
1^st^ trim. Nausea	44/205	48/205	0.70	30/148	32/148	0.88	14/57	16/57	0.82
	(21.5)	(23.4)		(20.3)	(21.6)		(24.6)	(28.1)	
Hospital admission because of hyperemesis	5/204	0/204	0.06	2/147	0/147	0.50	3/57	0/57	0.25
	(2.5)	(0)		(1.4)	(0)		(5.3)	(0)	
Hypertensive disorders									
Pregnancy induced hypertension	39/204	17/204	0.002	25/147	13/147	0.05	14/57	4/57	0.02
	(19.1)	(8.3)		(17.0)	(8.8)		(24.6)	(7.0)	
Pre-eclampsia	24/204	13/204	0.07	15/147	8/147	0.21	9/57	5/57	0.29
	(11.8)	(6.4)		(10.2)	(5.4)		(15.8)	(8.8)	
HELLP syndrome	2/204	1/204	1	1/147	1/147	1	1/57	0/57	1
	(1.0)	(0.5)		(0.7)	(0.7)		(1.8)	(0)	
Abnormal Placentation									
Placenta praevia	4/204	6/204	0.69	4/147	6/147	0.69	0/57	0/57	1
	(2.0)	(2.9)		(2.7)	(4.1)		(0)	(0)	
Placental abruption	0/204	2/204	0.5	0/147	2/147	0.50	0/57	0/57	1
	(0)	(1.0)		(0)	(1.4)		(0)	(0)	
Preterm labour									
pPROM	7/204	10/204	0.61	2/147	5/147	0.38	5/56	5/56	1
	(3.4)	(4.9)		(1.4)	(3.4)		(8.9)	(8.9)	
Preterm labour	32/204	44/204	0.12	15/147	26/147	0.10	17/57	18/57	1
	(15.7)	(21.6)		(10.2)	(16.7)		(29.8)	(31.6)	
Gestational diabetes	15/204	7/204	0.10	11/147	4/147	0.07	4/57	3/57	1
	(7.4)	(3.4)		(7.5)	(2.7)		(7.0)	(5.3)	
Cholestasis	1/205	2/205	1	0/147	1/147	1	1/57	1/57	1
	(0.5)	(01.0)		(0)	(0.7)		(1.8)	(1.8)	

Paired t tests for continuous variables and McNemar test for categorical variables.

*DO* Donated oocyte; *AO* Autologous oocyte.

Categorical variables are presented as numerator and denominator values (%), and continuous variables as mean (SD, standard deviation) for each group of interest.

### Infant outcome after oocyte donation versus autologous matched controls

Gestational age, birth weight, height and head circumference and the calculated standard deviations scores were comparable for both groups (Table 3). The children of oocyte recipients were not at increased risk for low-birth-weight (LBW), very-low-birth weight (VLBW) or poor Apgar scores. A higher incidence of preterm birth was observed in singletons and twin pregnancies after oocyte donation: 14.2% vs. 12.2% (*P* value for McNemar test <0.001) and 64.9% vs. 57.1% (*P* value for McNemar test <0.001), respectively. However, the mean gestational age, as well as the delivery rate before 34 weeks was not different.

**Table 3:** Infant outcome in pregnancies with donated oocytes versus individually matched pregnancies with autologous oocytes

	**All pregnancies**			**Singletons**			**Twins**		
	**DO**	**AO**	**P**	**DO**	**AO**	**P**	**DO**	**AO**	**P**
Live births	262	262		148	148		114	114	
Gestational age									
Mean gestational age	37.29	37.34	0.82	38.72	38.74	0.94	35.40	35.49	0.50
(SD)	(3.1)	(2.9)	(n = 259)	(2.3)	(2.0)	(n = 147)	(2.9)	(2.8)	(n = 112)
< 37 weeks of gestation	95/262	82/259	<0.001	21/148	18/147	<0.001	74/114	64/112	<0.001
(%)	(36.3)	(31.7)		(14.2)	(12.2)		(64.9)	(57.1)	
< 34 weeks of gestation	16/237	20/237	0.60	4/145	4/145	1	12/92	16/92	0.54
(%)	(6.8)	(8.4)		(2.8)	(2.8)		(13.0)	(17.4)	
Birth weight									
Mean birth weight	2832.9	2805.3	0.56	3211.3	3183.4	0.67	2321.2	2294.1	0.70
(SD)	(709.9)	(711.1)	(n = 254)	(593.5)	(583.4)	(n = 146)	(504.7)	(524.9)	(n = 108)
Birth weight	87/256	77/260	0.28	13/147	14/147	0.83	74/109	63/113	0.11
< 2500 g (%)	(34)	(29.6)		(8.8)	(9.5)		(67.9)	(55.8)	
Birth weight	7/256	11/260	0.48	3/147	2/147	1	4/109	9/109	0.27
< 1500 g (%)	(2.7)	(4.2)		(2)	(1.4)		(3.7)	(8)	
Mean birth length	48.1	48.1	1	49.7	49.6	0.81	45.7	45.8	0.795
(SD)	(3.6)	(3.5)	(n = 233)	(2.9)	(2.9)	(n = 138)	(3.2)	(3.0)	(n = 95)
Mean head circumference	33.6	33.4	0.31	34.2	34.2	0.95	32.7	32.1	0.10
(SD)	(2.3)	(2.2)	(n = 186)	(2.2)	(2.0)	(n = 113)	(2.1)	(2.0)	(n = 73)
APGAR scores									
1 minute APGAR scores									
Less than 4	3/224	5/233	0.73	2/132	1/133	1.00	1/92	4/100	0.63
	(1.3)	(2.1)	(n = 201)	(1.5)	(0.8)	(n = 120)	(1.1)	(4.0)	(n = 40)
Less than 7	30/224	26/233	0.46	19/132	10/133	0.076	11/92	16/100	0.34
	(13.4)	(11.2)	(n = 201)	(14.4)	(7.5)	(n = 120)	(12.0)	(16.0)	(n = 40)
5 minute APGAR scores									
Less than 4	0/224	1/230	1.00	0/132	0/131	NA	0/92	1/99	1.00
	(0)	(0.4)	(n = 201)	(0)	(0)		(0)	(1.0)	(n = 41)
Less than 7	4/224	4/230	1.00	3/132	1/131	0.63	1/92	3/99	1.00
	(1.8)	(1.7)	(n = 201)	(2.3)	(0.8)	(n = 118)	(1.1)	(3.0)	(n = 41)
10 minute APGAR scores									
Less than 4	0/217	0/194	NA	0/131	0/118	NA	0/86	0/76	NA
	(0)	(0)		(0)	(0)		(0)	(0)	
Less than 7	0/217	2/194	0.50	0/131	1/118	1.00	0/86	1/75	1.00
	(0)	(1.0)	(n = 163)	(0)	(0.8)	(n = 105)	(0)	(1.3)	(n = 29)

Paired t tests for continuous variables and McNemar test for categorical variables.

DO: Donated oocyte; AO: Autologeous oocyte.

Categorical variables are presented as numerator and denominator values (%), and continuous variables as mean (SD, standard deviation) for each group of interest.

NA, not applicable.

### Mode of delivery

Patients who conceived with donor oocytes were more likely to deliver by caesarean section (Table 4; singletons 50.0% vs. 37.2%: *P* = 0.06; twins 84.2% vs. 68.4%; *P* = 0.08). The significantly higher incidence of caesarean sections appears to be associated with a higher rate of non-elective, rather than elective caesarean sections. Women pregnant with autologous oocytes were more likely to undergo induction of labour and were also more likely to deliver spontaneously (Table 4).

**Table 4:** Induction of labour and mode of delivery

	**All pregnancies**			**Singletons**			**Twins**		
	**DO (%)**	**AO (%)**	**p**	**DO (%)**	**AO (%)**	**P**	**DO (%)**	**AO (%)**	**P**
									
Induction of labour (%)	16/201 (8.0)	41/201 (20.4)	<0.001	16/144 (11.1)	36/144 (25.0)	0.004	0/57 (0)	5/57 (8.7)	0.06
Mode of delivery									
Spontaneous delivery (%)	53/202 (26.2)	84/202 (41.6)	0.002	46/145 (31.7)	67/145 (46.2)	0.02	7/57 (12.3)	17/57 (29.8)	0.04
Assisted vaginal delivery (%)	30/202 (14.9)	25/202 (12.4)	0.56	28/145 (37.8)	24/145 (26.4)	0.65	2/57 (3.5)	1/57 (1.8)	1
Forceps* (%)	9/30 (30)	5/25 (20)		9/28 (32.1)	4/24 (16.7)		0/2 (0)	1/1 (100)	
Ventouse* (%)	21/30 (70)	20/25 (80)		19/28 (67.9)	20/24 (83.3)		2/2 (100)	0/1 (0)	
Caesarean section (%)	119/202 (58.9)	93/202 (46.0)	0.01	71/145 (50.0)	54/145 (37.2)	0.06	48/57 (84.2)	39/57 (68.4)	0.08
Elective c. Section (%)	55/119 (46.2)	61/93 (65.6)		31/71 (43.7)	33/54 (61.1)		24/48 (50.0)	28/39 (71.8)	

Paired t tests for continuous variables and McNemar test for categorical variables.

DO: Donated oocyte; AO: Autologeous oocyte.

Categorical variables are presented as numerator and denominator values (%), and continuous variables as mean (SD, standard deviation) for each group of interest.

*Forceps and Ventouse deliveries as proportion of all assisted vaginal deliveries.

** Elective caesarean sections as proportion of all caesarean sections.

### Multivariate logistic regression analysis

After testing all the parameters in the multivatiate regression model, after adjusting for paternal age, donor age, the number of embryos transferred, and singleton/twin pregnancy, the only factors that remained statistically significant were first trimester vaginal bleeding and PIH, with matched odds ratios (95% confidence limits) of 1.49 (1.04-2.15) and 1.50 (1.02-2.21), respectively (Table 5).

**Table 5:** Obstetrical outcome in pregnancies with donated oocytes versus individually matched pregnancies with autologous oocytes further adjusted for paternal age, oocyte age, number of embryos per transfer, and singleton/twin pregnancy

**Obstetrical outcome parameter**	**Matched OR**	**95% CI**
Vaginal bleeding		
1^st^ trimester	1.47	1.02 – 2.12
2^nd^ trimester	1.27	0.52 – 3.12
3^rd^ trimester	0.98	0.24 – 3.99
Nausea and vomiting		
1^st^ trimester nausea	0.87	0.58 -1.29
Hospital admission because of hyperemesis	1.88	0.59 – 6.04
Hypertensive disorders		
Pregnancy induced hypertension	1.50	1.02 – 2.19
Pre-eclampsia	1.31	0.83 – 2.08
Preterm labour		
pPROM	0.78	0.29 – 2.13
Preterm labour	0.74	0.47 – 1.16
Gestational diabetes	1.59	0.85 – 2.98

## Discussion

Our analysis provides evidence that oocyte donation is associated with an increased risk of PIH and first trimester vaginal bleeding. Although oocyte donation cycles appear to be associated with relatively high incidences of preeclampsia, gestational diabetes, preterm delivery, caesarean section and incidences of labour induction their incidences were not significantly increased in this study.

To our knowledge this is the largest cohort study examining the effect of oocyte donation on obstetric and perinatal outcomes including literally more patients than all the previous controlled trials cumulatively [12]. Furthermore, this study includes adequate matching for confounding factors, which was facilitated by a large prospectively collected ART outcome database. Another strength of the study is that both cohorts of pregnancies deriving from donor and autologous oocytes were matched, not only for age parity and plurality as previous trials did [12], but also for maternal ethnicity which may be a factor associated with the incidence of preeclampsia [13-15].

Previous studies that compared the obstetrical outcomes after oocyte donation and after standard in-vitro fertilisation (IVF) in patients of the same age group [16,17] have also reported an increased incidence of PIH. Söderström et al. found a significantly higher incidence of hypertensive disorders in the oocyte recipients although these individuals also had a significantly higher incidence of primiparity. Recently, the obstetrical outcome after oocyte donation compared with a selected control group, matched for potential confounding parameters, was the focus of research in three observational studies [18-20]. Nevertheless, in all three studies, the average age of individuals in the control groups was significantly lower than the age of the oocyte recipients. Several trials have shown that the incidence of preeclampsia is increased in oocyte recipients; although we did find an increase of 5.4% in oocyte recipient this was not significant (p = 0.07). This could be attributed to the fact that we matched for all potential confounding factor, including ethnicity. The only study that has similar design is the trial by Klatsly et al. (2010); nonetheless, our trial literally outnumbers this trial by 2.5 times including more than 400 patients in total.

Oocyte donation treatment confers a unique group of pregnancies partly because this method potentially extends biological fertility to ages well beyond the average age of menopause [21,22]. It is therefore important, but difficult, to identify an adequate control population without multiple confounding factors. Since nationwide US epidemiological data suggest that the risk of pre-eclampsia increases by 30% for every additional year beyond the age of 34 [23] we decided to match cases and controls according to their age. The primary purpose of the current study was to investigate the effect of donation; therefore, individual case–control matching also aimed to control for the confounding effect of parity, as nulliparity almost triples the risk for pre-eclampsia (2.91, 1.28 to 6.61) [24]. A possible limitation to the present study is the limited registration of the maternal risk factors BMI (54.1%) and smoking behaviour (45.6%). However, the available data showed that the incidence was similar in the two groups. Because no match was found for all OD pregnancies in women older than 43 years of age our comparative analysis do not allow to provide any statement regarding obstetrical or birth outcomes for OD pregnancies in this older age group.

Although several authors reported higher incidences of first trimester bleeding in ART pregnancies as compared to spontaneous pregnancies, it still remained unclear whether oocyte donation and the underlying mechanism leading to first trimester bleeding conferred two independent risk factors [25]. One study compared pregnancy outcomes after oocyte donation (n = 51) with those after IVF pregnancies and observed a significant increase of first trimester bleeding [17]. However, two further studies (n = 50; n = 71) did not observe a difference between these two populations [18,20]. In the current study (n = 205) we report a significant increase in first trimester bleeding (OR: 1.49; 95CI 1.036-2.150) in comparison with a matched control group.

Ongoing spontaneous pregnancies complicated by first-trimester bleeding confer an increased risk for preterm birth and lower birth weight [26,27]. De Sutter et al. found that the higher incidence of first-trimester bleeding was also associated with an increased risk for pregnancy complications in pregnancies following ART [28]. The study reported a lower mean duration of pregnancy and lower birth weight in pregnancies complicated with first trimester vaginal bleeding. However, in our series, the significant increase in first trimester bleeding did not negatively affect the perinatal outcome. The reported higher incidence of first trimester bleeding in ongoing OD pregnancies may possibly be explained by a reduced miscarriage vulnerability to first trimester bleeding. However, this hypothesis cannot be confirmed by the our data, as the matched controls were selected from pregnancies reaching 20 weeks of gestation aimed at evaluating the neonatal outcome.

First-trimester vaginal bleeding may indicate underlying placental dysfunction as it is associated with increased risks of preeclampsia or placental abruption [27]. Our study did not demonstrate an increased risk for any of these obstetrical complications in pregnancies complicated with first trimester bleeding.

As all donated oocytes in our centre undergo fertilisation by intra cytoplasmic sperm injection (ICSI] we selected control patients from our ICSI population. A meta-analysis of eight studies found that the risk for pre-eclampsia was significantly increased in women undergoing ART, with odds ratio of 1.55 (95% CI 1.23-1.95) [29]. Our data show that oocyte donation appears not to further increase that risk. Four meta-analyses concluded that compared with spontaneously conceived singletons, pregnancies after assisted fertilisation are more likely to be complicated by prematurity or low birth weight [29-32]. However, a large population-based cohort study comparing birth weight and gestational age between siblings born to women who had conceived both spontaneously and after ART concluded that the observed increased risks are attributable to the factors leading to infertility, rather than to the reproductive technology itself [33].

Conditional logistic regression was performed adjusted for paternal age, donor age and the number of embryos transferred. The influence of paternal genes on normal development and function of the placenta in human is known [34]. Paternal age is considered as a possible risk factor because of a potential mechanism leading to placental dysfunction associated with first trimester bleeding, PIH and preeclampsia [35]. Moreover, there are data suggesting a link between paternal age and placental abruption [36]. The mean age of the partner did not differ between pregnancies with donated versus autologous oocytes and further statistical analysis did not demonstrate any correlation between paternal age and adverse outcome in our series. Although both OD and control pregnancies were conceived after ICSI fertilisation, the indication for the ICSI technique differs. The inability to assess the impact of severe male infertility in the control population is a limitation to the study as this factor is known to affect pregnancy outcome.

Hypertensive disorders in pregnancy have a higher incidence in women of advanced age [37,38]. As the ages of the oocyte donor and the recipient differ significantly in an oocyte donation population, the logistic regression analysis was further adjusted for age of the oocyte donor in order to assess its possible influence. The age of the oocyte did not appear to influence the obstetrical or perinatal outcome.

Meta-analyses have reported that singleton children born after ART have an approximately twofold risk of being born preterm [29-31]. We observe similarly high incidences of pre-term birth in pregnancies after oocyte donation and in ICSI pregnancies (14.2% versus 12.2%).

As previously reported, a significantly higher incidence of delivery by caesarean section is observed in OD pregnancies. The higher incidence of caesarean sections appears to be associated with a higher proportion of non-elective, rather than elective caesarean sections. In spite of a significantly lower incidence of induction of labour, OD pregnancies are significantly more likely to end by a non-elective caesarean section. It has been argued that a higher level of concern underlies the observed increased incidence of caesarean birth [4,5,39]. However, ICSI pregnancies with autologous oocytes have also been associated with a higher level of concern [40].

## Conclusion

In summary, our study demonstrates that oocyte donation treatment is associated with an increased risk of first trimester bleeding and pregnancy induced hypertension in oocyte recipients. A major strength of our approach is that we largely controlled for known and important confounding factors. Although the obstetrical and perinatal outcomes are satisfactory, caution is mandatory in women with other risk factors undergoing egg donation.

## Competing interests

The authors declare that they have no competing interests.

## Authors’ contribution

DS idea, design and interpretation of data, writing of paper. MB statistical analysis, data collection. PH statistical analysis, interpretation of data. NPP interpretation of data, critical revision. MDV critical revision. GV critical revision. MC critical revision. PD idea, interpretation of data, approval of final version of paper. All authors read and approved the final manuscript.

## Supplementary Material

Additional file 1 **Table S1.** Obstetrical and infant outcome in oocyte recipients. Click here for file
