# Supplementary material for: Obstetric outcome in donor oocyte pregnancies: a matched-pair analysis
Source: Reprod Biol Endocrinol. 2012 Jun 6;10:42. doi: 10.1186/1477-7827-10-42 (PMC3488499; doi:10.1186/1477-7827-10-42)
Supplement: Additional file 1 — Table S1. Obstetrical and infant outcome in oocyte recipients. [file 1477-7827-10-42-S1.doc]

**Table S1**: Obstetrical and infant outcome in oocyte recipients

|  | **All (%)** | **Singletons (%)** | **Twins (%)** | **P** |
| --- | --- | --- | --- | --- |
| Obstetrical outcome |  |  |  |  |
| Number of pregnancies | 294 | 205 | 89 |  |
| Vaginal bleeding |  |  |  |  |
| 1^st^ trimester | 17.7 | 18.6 | 15.9 | 0.61 |
| 2^nd^ trimester | 2.3 | 3.4 | 2.6 | 0.69 |
| 3^rd^ trimester | 1.1 | 1.1 | 1.1 | 1 |
| Nausea and vomiting |  |  |  |  |
| 1^st^ trim. nausea | 20.8 | 19.2 | 23.9 | 0.42 |
| Hospital admission because of hyperemesis | 2.3 | 1.1 | 4.5 | 0.10 |
| Hypertensive disorders |  |  |  |  |
| Pregnancy induced hypertension | 19.2 | 15.3 | 27.3 | 0.03 |
| Pre-eclampsia | 13.2 | 9.6 | 20.5 | 0.02 |
| HELLP syndrome | 1.1 | 0.6 | 2.3 | 0.26 |
| Abnormal Placentation |  |  |  |  |
| Placenta praevia | 1.9 | 2.3 | 1.1 | 1 |
| Placental abruption | 0.4 | 0 | 1.1 | 0.33 |
| Preterm labour |  |  |  |  |
| pPROM | 5.3 | 1.1 | 13.6 | <0.001 |
| Preterm labour | 18.5 | 10.7 | 34.1 | <0.001 |
| Gestational diabetes | 7.5 | 7.3 | 8.0 | 1 |
| Cholestasis | 0.8 | 0 | 2.3 | 0.11 |
| Infant outcome |  |  |  |  |
| Gestational age |  |  |  |  |
| mean gestational age (weeks) (SD) | 37.3 (0.2) | 38.5 (2.5) | 35.3 (3.0) | <0.001 |
| < 37 weeks of gestation (%) | 41.3 (157/380) | 17.6 (36/205) | 69.1 (121/175) | <001 |
| < 34 weeks of gestation (%) | 12.6 (48/380) | 4.4 (9/205) | 22.3 (39/175) | <0.001 |
| Birth weight |  |  |  |  |
| Mean birth weight (gram) (SD) | 2781.3 (741.1) | 3167.5 (659.8) | 2312.2 (536.1) | <0.001 |
| Birth weight < 2500 g (%) | 129/372 (34.7) | 22/204 (10.8) | 107/168 (63.7) | <0.001 |
| Birth weight < 1500 g (%) | 17/372 (4.6) | 6/204 (2.9) | 11/168 (6.5) | 0.13 |
| Mean birth length (SD) | 47.76 (3.8) | 49.3 (3.5) | 45.8 (3.2) | <0.001 |
| Mean head circumference (SD) | 38.3 (3.6) | 39.0 (3.6) | 37.3 (3.6) | 0.69 |
| APGAR scores |  |  |  |  |
| 1 minute APGAR score < 4 | (2.2) 7/315 | (2.8) 5/181 | (1.5) 2/134 | 0.70 |
| 1 minutes APGAR score < 7 | (14.9) 47/315 | (14.4) 26/181 | (15.7) 21/134 | 0.75 |
| 5 minutes APGAR score < 4 | (0) 0/317 | (0) 0/181 | (0) 0/136 | NA |
| 5 minutes APGAR score < 7 | (2.2) 7/317 | (2.8) 5/181 | (2.0) 2/136 | 0.71 |
| 10 minutes APGAR score < 4 | (0) 0/304 | (0) 0/178 | (0) 0/126 | NA |
| 10 minutes APGAR score < 7 | (0.3) 1/304 | (0.6) 1/178 | (0) 0/126 | 1.00 |
| Sex (% male) | 51.1 | 46.1 | 56.9 | 0.04 |

Student t test for continuous variables and Fisher’s exact test for categorical variables
